# Genetic characteristics, antimicrobial susceptibility, and virulence genes distribution of *Campylobacter* isolated from local dual-purpose chickens in central China

**DOI:** 10.3389/fcimb.2023.1236777

**Published:** 2023-09-07

**Authors:** Jia Xiao, Yiluo Cheng, Wenting Zhang, Qin Lu, Yunqing Guo, Qiao Hu, Guoyuan Wen, Huabin Shao, Qingping Luo, Tengfei Zhang

**Affiliations:** ^1^ Key Laboratory of Prevention and Control Agents for Animal Bacteriosis (Ministry of Agriculture and Rural Affairs), Institute of Animal Husbandry and Veterinary, Hubei Academy of Agricultural Sciences, Wuhan, China; ^2^ Hubei Provincial Key Laboratory of Animal Pathogenic Microbiology, Institute of Animal Husbandry and Veterinary, Hubei Academy of Agricultural Sciences, Wuhan, China; ^3^ Institute of Animal Husbandry and Veterinary, Hubei Academy of Agricultural Sciences, Hubei Hongshan Laboratory, Wuhan, China

**Keywords:** *Campylobacter*, antibiotic resistance, whole-genome sequencing, antibiotic-resistance gene, virulence factor

## Abstract

Food-borne antibiotic-resistant *Campylobacter* poses a serious threat to public health. To understand the prevalence and genetic characteristics of *Campylobacter* in Chinese local dual-purpose (meat and eggs) chickens, the genomes of 30 *Campylobacter* isolates, including 13 C*. jejuni* and 17 C*. coli* from Jianghan-chickens in central China, were sequenced and tested for antibiotic susceptibility. The results showed that CC-354 and CC-828 were the dominant clonal complexes of *C. jejuni* and *C. coli*, respectively, and a phylogenetic analysis showed that three unclassified multilocus sequence types of *C. coli* were more closely genetically related to *C. jejuni* than to other *C. coli* in this study. Of the six antibiotics tested, the highest resistance rates were to ciprofloxacin and tetracycline (100%), followed by lincomycin (63.3%), erythromycin (30.0%), amikacin (26.7%), and cefotaxime (20.0%). The antibiotic resistance rate of *C. coli* was higher than that of *C. jejuni*. The GyrA T86I mutation and 15 acquired resistance genes were detected with whole-genome sequencing (WGS). Among those, the GyrA T86I mutation and *tet(O)* were most prevalent (both 96.7%), followed by the *blaOXA*-type gene (90.0%), *ant(6)-Ia* (26.7%), *aac(6’)-aph(3’’)* (23.3%), *erm*(B) (13.3%), and other genes (3.3%). The ciprofloxacin and tetracycline resistance phenotypes correlated strongly with the GyrA T86I mutation and *tet(O)*/*tet(L)*, respectively, but for other antibiotics, the correlation between genes and resistance phenotypes were weak, indicating that there may be resistance mechanisms other than the resistance genes detected in this study. Virulence gene analysis showed that several genes related to adhesion, colonization, and invasion (including *cadF*, *porA*, *ciaB*, and *jlpA*) and cytolethal distending toxin (*cdtABC*) were only present in *C. jejuni*. Overall, this study extends our knowledge of the epidemiology and antibiotic resistance of *Campylobacter* in local Chinese dual-purpose chickens.

## 1 Introduction

According to the report of the World Health Organization (WHO), food-borne diseases, ranging from diarrhea to cancer, are a major cause of human morbidity and mortality and affect one in 10 people worldwide every year (WHO, 2022). Campylobacteriosis is one of the most frequently reported food-borne diseases throughout the world (EFSA BIOHAZ Panel [EFSA Panel on Biological Hazards] et al., 2020). The acute infectious diarrhea caused by *Campylobacter* is mainly treated with antibiotics, such as fluoroquinolones and macrolides (Pham et al., 2016). However, the use of antibiotics in both human treatments and animal breeding hisolated from poultry meat samples.as caused antimicrobial resistance in *Campylobacter* to become an increasingly serious problem, and has posed a serious threat to public health over the past two decades (Luangtongkum et al., 2009). In 2017, fluoroquinolone-resistant *Campylobacter* was listed as one of the six high-priority antimicrobial-resistant pathogens by WHO (Romanescu et al., 2023). In China, bacterial antibiotic resistance monitoring data show that *Campylobacter* has maintained a high level of resistance to ciprofloxacin (> 90%) in various regions, (Li et al., 2016; Wang et al., 2016; Ju et al., 2018).

Poultry is the most important natural host of *Campylobacter*. In the European Union, the average prevalence of *Campylobacter* in birds and contaminated broiler carcasses is 71.2% and 75.8%, respectively (Soro et al., 2020), and more than 90% of commercial laying hens are colonized with *Campylobacter* (Jones et al., 2016). The breed of chicken is directly related to *Campylobacter* infection. Brena (Brena, 2013) reported that chickens reared indoors under higher welfare standards with decreased stocking density, the prevalence of *Campylobacter* was lower in a slower-growing breed (Hubbard JA57) than in a standard fast-growing breed (Ross 308). However, Humphrey et al. (Humphrey et al., 2014) demonstrated no intrinsic difference in the susceptibility of broiler breeds to *C. jejuni* under their experimental conditions.

China has many indigenous poultry resources, and many local chickens are dual-purpose (meat–egg) producers, with a longer growth cycle than broiler chickens. In general, traditional commercial broilers, such as AA broiler, Ross 308, are slaughtered in about 42 days (Fortuoso et al., 2019). However, some of the Chinese local chickens, such as Jianghan-chickens, usually start laying eggs at 140-150 days and then are slaughtered as food around 300 days. The life cycle of this type of production differs from that of commercial chickens, which may make the ecology (including antibiotic resistance) of *Campylobacter* different in production cycle. Previous studies have reported that under the same breeding conditions, the Huainan partridge chicken had a lower rate of *Campylobacter* infection than Heihua chickens or Ni-ke hon chickens, but a higher rate than AA+ chickens (Huang et al., 2009). Bai et al. (Bai et al., 2021) found that the isolation rate of *Campylobacter* was lower in slaughterhouses processing yellow feather broilers (14.2%) than in those processing white feather broilers or turkeys (from 26.3 to 100%). However, there are still few data on the prevalence of *Campylobacter* in local chickens in China.

The prevalence of antibiotic-resistant *Campylobacter* in poultry also cannot be ignored. Bacteria usually acquire antimicrobial resistance (AMR) by two main pathways. One involves chromosomal mutations at the target sites of antibiotic action, such as the point mutation in the *gyrA* gene that causes resistance to fluoroquinolone antibiotics (Iovine, 2013). The second involves the horizontal gene transfer of mobile genetic elements that contain resistance genes (Aksomaitiene et al., 2021). In the past few years, antibiotic-resistant *Campylobacter* in chicken house environment, eggshell, carcasses, poultry production, and the processing chain have been reported in many countries (Modirrousta et al., 2016; Tang et al., 2020b; Habib et al., 2023). Although several studies have detected antimicrobial-resistant *Campylobacter* in dual-purpose chickens (Foster-Nyarko et al., 2021; Metreveli et al., 2022; Rangaraju et al., 2022), Jianghan-chicken is a unique resource, which distributed in Central China. At present, the research on Jianghan-chicken is mainly focused on the eradication of *Salmonella* pullorum and avian leukosis, the overall resistance and virulence of *Campylobacter* in this chicken are unclear. Notably, the prevalence of *Campylobacter*, the generation and spread of its antibiotic resistance, and the complexity of its pathogenesis are probably related to the diversity of the *Campylobacter* genome. Many virulence genes have undergone expansion or contraction in specific lineages, resulting in differences in the content of virulence genes and ultimately leading to the specificity of their pathogenicity (Zhong et al., 2022). Fortunately, DNA sequencing technologies provide efficient methods with which to understand the antibiotic-resistance and pathogenic mechanisms of *Campylobacter*.

In this study, we investigated the genetic diversity, antibiotic resistance, and the distributions of the resistance and virulence genes of *Campylobacter* in local dual-purpose Jianghan-chickens in four regions of central China. We also used whole-genome sequencing (WGS) to evaluate the genetic diversity of *Campylobacter* and the phenotypic and genetic determinants associated with its intrinsic resistance. This data from this study extends our understanding of the prevalence and genomic characteristics of food-borne *Campylobacter* in local chickens in China.

## 2 Materials and methods

### 2.1 Bacterial isolates and culture conditions

In this study, 30 *Campylobacter* isolates were isolated from 312 samples collected from eight chicken farms breeding local dual-purpose (meat–egg) chickens in four regions of central China in 2022 (**Supplementary Table 1**). Freshly collected cloacal swabs were stored in Cary–Blair Modified Transport Medium (Amresco, Englewood, USA) and transported to the laboratory at 4°C for *Campylobacter* isolation. The samples were pre-enriched in Bolton broth containing *Campylobacter* growth supplement (Oxoid, Basingstoke, UK) and *Campylobacter* Bolton broth selective supplement (Oxoid), and cultured at 42°C for 24 h under microaerobic conditions (5% O_2_, 10% CO_2_, and 85% N_2_). Subsequently, 100 µl cultures were inoculated on modified charcoal cefoperazone deoxycholate agar (mCCDA, Oxoid) plates containing *Campylobacter* CCDA selective supplements at 42°C under microaerobic condition for 48 h. Suspected positive colonies were identified with Gram staining and 16S rDNA PCR (Linton et al., 1997). All isolates were identified with PCR targeting the *C. jejuni*-specific *hipO* gene and the *C. coli*-specific *asp* gene (Lawson et al., 1998).

### 2.2 Antimicrobial sensitivity testing

All isolates were tested for antimicrobial susceptibility to ciprofloxacin, tetracycline, cefotaxime, amikacin, erythromycin, and lincomycin with the disk diffusion method on Mueller Hinton Agar (Oxoid), according to the Clinical and Laboratory Standards Institute (CLSI) guidelines (Igwaran and Okoh, 2020). When the isolates were resistant to at least three different types of antibiotics, they were considered multidrug resistant (MDR). *Escherichia coli* ATCC 25922 was used as a quality control strain.

### 2.3 Whole-genome sequencing and analysis

The genomic DNA of the *Campylobacter* species was extracted with the TIANamp Bacteria DNA Kit (Tiangen, Beijing, China). The purity and concentration of the genomic DNA were determined by NanoDrop™ One sectrophotometer (Thermo Fisher Scientific, Waltham, MA, USA). Genomic DNA (5 μg; OD_260/280 = _1.8–2.0) was used for library construction. The Illumina NovaSeq 6000 sequencing platform (MajorBio Co., Shanghai, China) was used to sequence those libraries with a 2 × 150-bp read length. The raw reads obtained after sequencing were filtered with the fastp software (version 0.19.6) (Chen et al., 2018) and clean reads were obtained after the adapter sequences and low-quality sequences (Q < 20) were removed. The clean reads were then assembled with SOAPdenovo version 2.04 (Luo et al., 2012). The assembled contigs were uploaded to PubMLST (https://pubmlst.org/Campylobacter/) to determine their multilocus sequence types (STs) and clonal complexes (CCs). The phylogenetic tree and SNP count matrix heat map based on SNP analysis was obtained by using the online tool “multiple genome analysis” provided by BacWGSTdb 2.0 (http://bacdb.cn/BacWGSTdb/). RM1221_CP000025, which had abundant studies on its genome (Parker et al., 2006; Neal-McKinney et al., 2021; St. Charles et al., 2022), was selected as reference genome in BacWGSTdb tool and the construction of the phylogenetic tree in this tool relies on Neighbor-Joining (NJ) algorithm (Feng et al., 2021). The virulence genes were predicted based on the Virulence Factor Database (VFDB; http://www.mgc.ac.cn/VFs/). The tool ResFinder v.4.1 was used to detect acquired AMR genes and point mutations in specific genes conferring AMR; 90% minimum percentage identity and 60% minimum length coverage were used as the selection criteria. The sequence of the regulatory region of the *cmeABC* promoter (CmeR-Box) which is a 16-base inverted repeat sequence [TGTAATA (or T) TTTATTACA] (Cheng et al., 2020) and the amino acid sequence of CmeR were obtained by comparing the sequence alignment through BLAST (https://blast.ncbi.nlm.nih.gov/Blast.cgi). RAST Server (Rapid Annotation using Subsystem Technology) was used for Genome annotation of the assembled genome of multi-drug resistant *Campylobacter spp*, and the annotation scheme was ClassicRAST (http://rast.theseed.org/FIG/rast.cgi). Antibiotic resistance gene were also analyzed by Mobile Element Finder (https://cge.food.dtu.dk/services/MobileElementFinder/), and SnapGene® 2.3.2 was used to visualize gene arrangement.

### 2.4 Correlation analysis of susceptibility phenotypes and genotypes

The possible link between the *Campylobacter* resistance phenotype and the genotype predicted with WGS was analyzed by manually comparing the susceptibility test results (resistance or susceptibility) with the presence of known corresponding resistance genes and/or specific mutations. The percentage correlation between the resistance phenotype and genotype was calculated as the sum of true positives and true negatives divided by all the isolates tested. The positive predictive value was calculated by dividing the true positives by the sum of the true positives and false negatives, and the negative predictive value was calculated by dividing the true negatives by the sum of the true negatives and false positives. Sensitivity was calculated by dividing the true positives by the sum of the true positives and false positives, and specificity was calculated by dividing the true negatives by the sum of the true negatives and false negatives (Hodges et al., 2021).

## 3 Results

### 3.1 Genetic diversity analysis

**Figure 1:**
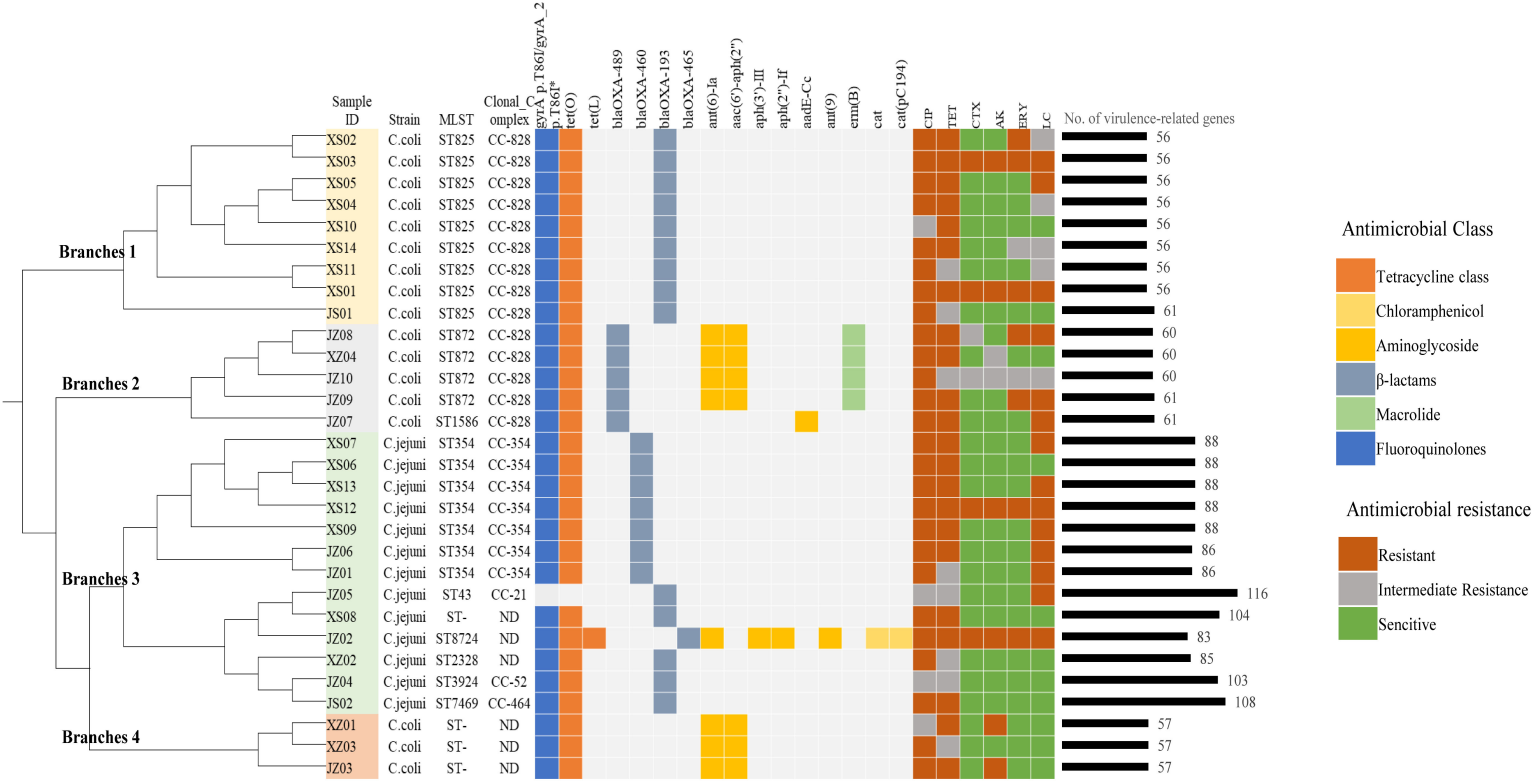
Genetic relationships, antimicrobial-resistance phenotypes, and the distributions of resistance- and virulence-related genes determined in this study. The phylogenetic tree was constructed based on genomic single-nucleotide polymorphisms, and the reference genome was RM1221_CP000025. The genetic determinants of antibiotic resistance are grouped according to their corresponding antibiotic categories and are color coded. The isolates were divided into 4 branches of the tree and distinguished by different colors: yellow (branch 1), gray (branch 2), green (branch 3), red (branch 4). CIP, ciprofloxacin; TET, tetracycline; CTX, cefotaxime; AK, amikacin; ERY, erythromycin; LC, lincomycin. *Point mutation.

Among the 30 *Campylobacter* isolates (13 C*. jejuni* and 17 C*. coli*) sequenced, nine sequence types (STs) in five clonal complexes (CC) based on multilocus sequence typing (MLST) were identified. Three *C. coli* and one *C. jejuni* isolates were not assigned an ST. ST8724 and ST2328 were not defined as a CC (**Figure 1**; **Supplementary Table 2**). The dominant ST for *C. jejuni* was ST354 (53.8%, 7/13), and the other isolates belonged to ST7469, ST8724, ST43, ST3924, or ST2328 (each 7.6%, 1/13). CC-354 was the dominant CC among the *C. jejuni* isolates. Among the *C. coli* isolates, ST825 was the most frequent ST (52.9%, 9/17), followed by ST872 (23.5%, 4/17) and ST1586 (5.9%, 1/17), and all of these assigned STs belonged to CC-828.

SNP analysis was further carried out, and we found that there was a certain genetic diversity among these isolates, and these differences involved SNP differences vary greatly, from a few to thousands (**Supplementary Figure 1**). All the isolates can be cluster to four main branches of the phylogenetic tree (**Figure 1**). Branches 1 and 2 contained the major clonal complex CC-828 of *C. coli*, and branch 3 contained the main clonal complex (CC-354) of *C. jejuni*. Interestingly, branch 4, which contained the three *C. coli* isolates with unassigned STs, clustered with the larger branch containing *C. jejuni*.

### 3.2 Antimicrobial susceptibility

All the isolates were tested for susceptibility to six antibiotics. As shown in **Table 1**, all showed resistance to ciprofloxacin and tetracycline (100% in both *C. jejuni* and *C. coli*). More than half the isolates were resistant to lincomycin (61.5% of *C. jejuni* and 64.7% of *C. coli*). The resistance rates of *Campylobacter* to erythromycin, amikacin, and cefotaxime were 30.0%, 26.7%, and 20.0%, respectively. The resistance rate of *C. coli* to erythromycin was 41.2%, which was more than twice that of *C. jejuni* (15.4%). The data showed similar trends for amikacin (35.3% in *C. coli* and 15.4% in *C. jejuni*). Among the 30 isolates, 22 were resistant to three or more classes of antimicrobial agents, and the most prevalent pattern of MDR was resistance to ciprofloxacin, tetracycline, and lincomycin (45.5%, 10/22) (**Figure 1**).

**Table 1:** Resistance rates of tested *Campylobacter* isolates to six antibiotics.

Antibiotic category	Antimicrobial Agent^a^	*C. jejuni* (n=13)		*C. coli* (n=17)		Total (n=30)	
		No. of resistant isolates^b^	Resistance rates (%)	No. of resistant isolates^b^	Resistance rates (%)	No. of resistant isolates^b^	Resistance rates (%)
Fluoroquinolones	CIP	13	100.0%	17	100.0%	30	100.0%
Tetracycline class	TET	13	100.0%	17	100.0%	30	100.0%
β-lactams	CTX	2	15.4%	4	23.5%	6	20.0%
Aminoglycosides	AK	2	15.4%	6	35.3%	8	26.7%
Macrolides	ERY	2	15.4%	7	41.2%	9	30.0%
Lincosamides	LC	8	61.5%	11	64.7%	19	63.3%

^a^CIP, Ciprofloxacin; TET, Tetracycline; CTX, Cefotaxime; AK, Amikacin; ERY, Erythromycin; LC, Lincomycin. ^b^Resistant isolates contain resistance and intermediate resistance.

### 3.3 Antibiotic resistance genes and resistance mutations

In this study, a C257T chromosomal point mutation in the *gyrA* gene, which conferring the Thr-86-Ile substitution, and 15 acquired resistance genes were identified by genome-wide analysis. **Figure 1** and **Supplementary Table 3** show the distributions of the genetic determinants of resistance detected in each isolate with WGS.

Of all the isolates tested, 96.7% (29/30) carried the *gyrA* gene point mutation (C257T) along with a ciprofloxacin resistance phenotype. The correlation analysis of resistance phenotype and genotype showed that the *gyrA* C257T mutation correlated strongly with ciprofloxacin resistance (100% in *C. coli* and 92.3% in *C. jejuni*) (**Table 2**).

**Table 2:** Correlation analysis of antibiotic resistance phenotype and antibiotic resistance determinants.

Antibiotic class	Antibiotic(s) tested by AST	Strains	Phenotype	No. of isolates	Resistance gene(s) or mutation(s) corresponding to resistance phenotype	No. with AMR gene present	Correlation between genotype and phenotype	Positive predictive values	Negative predictive value	Sensitivity	Specificity
Fluoroquinolones	CIP	*C. coli*	R/IR	17	GyrA_2p.T86I	17	100.0%	100.0%	–	100.0%	–
			S	0		0					
		*C. jejuni*	R/IR	13	GyrA T86I	12	92.3%	92.3%	–	100.0%	0.0%
			S	0		0					
Tetracycline class	TE	*C. coli*	R/IR	17	tet(O)	17	100.0%	100.0%	–	100.0%	–
			S	0		0					
		*C. jejuni*	R/IR	13	tet(O); tet(L)	12	92.3%	92.3%	–	100.0%	0.0%
			S	0		0					
β-lactams	CTX	*C. coli*	R/IR	4	blaOXA-489; blaOXA-193	4	41.2%	100.0%	23.1%	28.6%	100.0%
			S	13		10					
		*C. jejuni*	R/IR	2	blaOXA-465; blaOXA-460; blaOXA-193;	2	15.4%	100.0%	0.0%	15.4%	–
			S	11		11					
Aminoglycosides	AK	*C. coli*	R/IR	6	ant(6)-Ia; aac(6’)-aph(2’’); aadE-Cc	4	64.7%	66.7%	63.6%	50.0%	77.8%
			S	11		4					
		*C. jejuni*	R/IR	2	aph(2’’)-If; aph(3’)-III; ant(6)-Ia;ant(9)	1	92.3%	50.0%	100.0%	100.0%	91.7%
			S	11		0					
Macrolides	ERY	*C. coli*	R/IR	7	erm(B)	3	70.6%	42.9%	90.0%	75.0%	69.2%
			S	10		1					
		*C. jejuni*	R/IR	2	–	0	84.6%	0.0%	100.0%	–	84.6%
			S	11		0					
Lincosamides	LC	*C. coli*	R/IR	11	erm(B)	3	47.1%	27.3%	83.3%	75.0%	38.5%
			S	6		1					
		*C. jejuni*	R/IR	8	–	0	38.5%	0.0%	100.0%	–	38.5%
			S	5		0					

R, resistance; IR, intermediate resistance; S, sensitive.

Most of the isolates (96.7%, 29/30) contained the *tet(O)* gene, and one *C. jejuni* strain carried *tet(L)* (3.3%, 1/30). All isolates showed tetracycline resistance. The correlation between the tetracycline resistance phenotype and the resistance gene *tet*(*O*) or *tet(L*) was 100% in *C. coli* and 92.3% in *C. jejuni*.

The *blaOXA*-type β-lactamase-encoding gene was identified in 27 strains (90%, 27/30). And 22.2% (6/27) of isolates were resistant to cefotaxime.

Six aminoglycoside antibiotic resistance genes were detected in our isolates: *ant(6)-Ia* (26.7%, 8/30), *aac(6’)-aph(3’’)* (23.3%, 7/30), *aph(3’)-III* (3.3%, 1/30), *aph(2’’)-If* (3.3%, 1/30), *ant(9)* (3.3%, 1/30), and *aadE-Cc* (3.3%, 1/30). In this study, these genes mainly occurred in pairs, such as *ant(6)-Ia* and *aac(6’)-aph(3’’)*, in *C. coli* (23.3%, 7/30). These resistance gene combinations did not correlate strongly with the amikacin resistance phenotypic in *C. coli* (64.7%), but did correlate strongly with it in *C. jejuni* (92.3%).

The erythromycin and lincomycin resistance gene *erm*(B) was only identified in four *C. coli* isolates (13.3%, 4/30). The correlation between *erm*(B) and the erythromycin or lincomycin resistance phenotype was not strong (70.6% or 47.1%, respectively, in *C. coli*; and 84.6% or 38.5%, respectively, in *C. jejuni*). Further analysis of the isolates for point mutations in 23S rRNA revealed eight mutations in total (**Supplementary Table 4**), although neither the A2075G nor A2074C/G mutation, which reportedly cause erythromycin resistance, was detected.

### 3.4 CmeR-Box polymorphisms

A CmeR-Box polymorphism analysis of all isolates (**Table 3**) detected six CmeR-Box variants in 28 isolates. Among these, point substitutions were most common (96.4%), involving 17 C*. coli* and 10 C*. jejuni* isolates, whereas only one *C. jejuni* isolate (3.6%) had a point deletion, and no point insertion was detected in the CmeR-Box.

**Table 3:** CmeR-Box polymorphisms in *C. jejuni* and *C. coli* isolates.

	CmeR-Box polymorphisms^a^	No. of isolates	% of isolates
*C. jejuni*	TGTAATAAAAATTATA	6	20.0%
	TGTAATAAATATTATA	3	10.0%
	TGTGATAAAAATTACA	1	3.3%
	TGTAATAAA-ATTACA	1	3.3%
	TGTAATAAAAATTACA	2	6.7%
*C. coli*	TGTAATAAATATTACA	16	53.3%
	TGTAATAAATATTGCA	1	3.3%

^a^underline means point substitution, “-” means point deletion.

### 3.5 Genetic environment analysis of antibiotic resistance gene clusters in an MDR *C. jejuni* isolate

**Figure 2:**
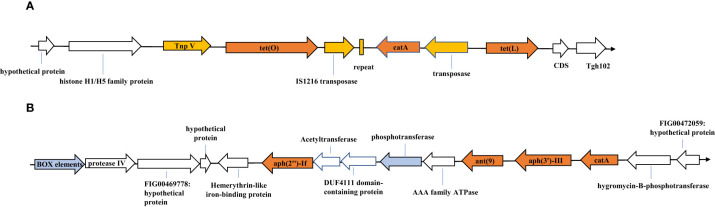
Resistance gene clusters identified in Campylobacter jejuni strain JZ02. **(A)** Tetracycline resistance gene cluster; **(B)** Aminoglycosides resistance gene cluster. SnapGene® 2.3.2 made this figure.

We analyzed the genetic environments of the resistance genes in *C. jejuni* JZ02, which was resistant to all of six antibiotics tested. Two antibiotic resistance gene clusters were detected (**Figure 2**). Gene cluster 1 contained the *tet(O)*, *tet(L)*, and *cat* (pC194) genes (**Figure 2A**). A transposase was encoded upstream from the *tet(L)* gene, and a 39-bp repeat and another transposase gene that shared 100% identity with IS1216 family transposase gene, were detected between *tet (O)* and *cat* (pC194). Moreover, a transposon encoding the protein TnpV was detected upstream from the *tet(O)* gene. Gene cluster 2 consisted of the *ant (9)*, *aph(3’)-III*, *aph(2’’)-If*, and *cat* genes (**Figure 2B**). However, no mobile genetic elements or repetitive sequences were detected in this gene cluster, although a box element and several hypothetical proteins with sequences similar to those of some Gram-positive bacteria were found.

### 3.6 Virulence gene detection

Based on the VFDB, 126 virulence-related genes, involving adhesion, invasion, motility, toxins, and the type IV secretion system, were identified (**Supplementary Table 5**). We observed more virulence-related genes in *C. jejuni* (83–116 per isolate) than in *C. coli* (56–61 per isolate), and among them, isolates *C. jejuni* JZ05 (CC-21) and JS02 (CC-464) had the most virulence-related genes (**Figure 1**). Most of the genes only detected in *C. jejuni* were related to motility and adhesion, including *cadF*, *htrB*, *pebA*, *ciaB*, *jlpA*, and *cheA*, and genes encoding cytolethal distending toxin (*cdtABC*) were also only detected in *C. jejuni*. Type IV secretion system genes, including *virB11*, *virB10*, *virB9*, *virB8*, *virB4*, and *virD4*, were detected in one *C. jejuni* isolate (3.3%), and *wlaN* was only found in two *C. jejuni* isolates (6.7%) (**Table 4**). *Campylobacter* isolates in most branches (branch 1, 2, 4) of the phylogenetic tree had similar numbers of virulence genes, and the categories of these genes were not quite different. Interestingly, in branch 3, the type and abundance of virulence genes vary greatly from different ST types, and most of the different genes are related to capsular synthesis and immune regulation. Some isolates with more virulence-related genes were distributed in a sub-branch of branch 3.

**Table 4:** Frequencies of parts of predicted virulence-related factors in the genomes of 30 *Campylobacter* isolates.

VF class	Related genes	*C. jejuni* (n=13)		*C. coli *(n=17)		Total (n=30)	
		NO.of isolate	% of isolates	NO.of isolate	% of isolates	NO.of isolate	% of isolates
Adherence	*cadF*	13	100.00%	0	0.00%	13	43.30%
	*porA*	5	38.50%	0	0.00%	5	16.70%
	*pebA*	13	100.00%	0	0.00%	13	43.30%
	*jlpA*	13	100.00%	0	0.00%	13	43.30%
Immune modulation	*htrB*	13	100.00%	0	0.00%	13	43.30%
	*wlaN*	2	15.40%	0	0.00%	2	6.70%
	*Cj1135*	13	100.00%	0	0.00%	13	43.30%
	*kpsD/M*	13	100.00%	17	100.00%	30	100.00%
	*neuA1*	2	15.40%	0	0.00%	2	6.70%
	*kpsF*	13	100.00%	14	82.40%	27	90.00%
	*cheA*	13	100.00%	0	0.00%	13	43.30%
Type IV secretion system	*virB10/virB11/virB4/virB8/virB9/virD4*	1	7.70%	0	0.00%	1	3.30%
Toxin	*cdtA*	12	92.30%	0	0.00%	12	40.00%
	*cdtB*	13	100.00%	0	0.00%	13	43.30%
	*cdtC*	13	100.00%	0	0.00%	13	43.30%
Motility	*flgB*	13	100.00%	17	100.00%	30	100.00%
	*flhB*	13	100.00%	17	100.00%	30	100.00%
	*flaA*	10	76.90%	6	35.30%	16	53.30%
Invasion	*ciaB*	13	100.00%	0	0.00%	13	43.30%

## 4 Discussion

*Campylobacter* is the main bacterial pathogen causing human diarrhea worldwide, and its increasing prevalence and antibiotic resistance have caused great concern globally in recent years, in both human and veterinary clinics. Poultry is the main host of *Campylobacter*, but the prevalence of the pathogen varies across different species and different regions. For instance, an investigation in southeastern Italy showed that the prevalence of *C. jejuni* was higher in broilers than in laying hens (45.7% and 21.1%, respectively) (Parisi et al., 2007). In Europe, the prevalence of broiler flocks colonized with *Campylobacter* ranged from 18% to > 90% in different countries (Newell and Fearnley, 2003). Meat–egg dual-purpose local chickens may differ from commercial varieties because their breeding modes and breeding cycles differ, and they may pose a potential risk of *Campylobacter* transmission to both meat and eggs (Ahmed et al., 2021). Therefore, we investigated the phylogenetic relationships, virulence genes, antibiotic resistance, and genetic bases of the resistance phenotypes of *Campylobacter* isolates collected from local meat–egg dual-purpose chicken in China.

In this study, we identified two main prevalent *Campylobacter* species, *C. jejuni* and *C. coli*, and found strong genetic diversity in the *Campylobacter* strains transmitted in these chickens. The National Center for Biotechnology Information (NCBI) database indicated that CC-354 strains occur mainly in the United States and the United Kingdom, whereas they are quite dispersed in other countries (Yu et al., 2020). A previous study showed that CC-353 and CC-464 are the dominant CCs of *C. jejuni* in central China, and CC-354 was the dominant population of *C. jejuni* detected in the present study. CC-21 is also the most frequently reported *C. jejuni* genotype in diarrhea patients in China (Zhang et al., 2020b), and in Zhang et al.’s study (Zhang et al., 2020a), CC-21 was also the dominant *Campylobacter* CC in chickens in southeastern China. However, in the present study, only one strain belonging to CC-21 was isolated, suggesting that the diversity of *C. jejuni* may vary by region and sample source, and that the epidemic patterns of *Campylobacter* may differ in local meat–egg dual-purpose chickens. Three ST types, ST1586, ST872, and ST828, were found in the *C. coli* isolates, which belong to the same clonal complex, CC-828. This was expected because CC-828 is the dominant population of *C. coli*, and a large number of past studies have reported its prevalence around the world (Zhang et al., 2016; Di Giannatale et al., 2019; Gomes et al., 2019). Based on principal component analysis (PCA) on the evolutionary distances of core gene families, Snipen et al. reported that *Campylobacter* has a mixed evolutionary pattern characterized by genomes (Snipen et al., 2012). It is noteworthy that the three *C. coli* strains with undefined STs detected in this study clustered with *C. jejuni* on the same large branch of a phylogenetic tree based on a genomic SNP analysis, suggesting that their genetic relationship was close. Previous studies have shown that an bidirectional increase in the rate of recombination between *C. jejuni* and *C. coli* has led to the gradual convergence of the two species (Sheppard et al., 2008).

Antibiotic resistance has become one of the most important factors threatening human public health globally (Mancuso et al., 2021). It is noteworthy that 73.3% of *Campylobacter* isolates were multidrug resistant in the present study. The resistance rates of *Campylobacter* to ciprofloxacin and tetracycline in China are high, and studies have reported rates of 90%–100% in broilers (Ma et al., 2014; Li et al., 2016; Wang et al., 2021). In the present study, there were similar high resistance rates to ciprofloxacin and tetracycline in both *C. jejuni* and *C. coli*. In the past, fluoroquinolones have been widely used in the edible animal industry, especially in poultry production, although tetracyclines are also commonly used to treat and prevent bacterial diseases in poultry in China. This may explain the high resistance rates to these two antibiotics in *Campylobacter*. In the early 1980s, the development and introduction of the third-generation extended-spectrum cephalosporin cefotaxime provided a new treatment for patients infected with Gram-negative bacilli (Hawkey, 2008). Here, we detected a relatively low rate of cefotaxime resistance (20.0%). Although it is not approved for use in food animals in China (Dai et al., 2008), we detected a high rate of amikacin resistance in *Campylobacter* (26.7%). Nor did the proportion of erythromycin-resistant isolates in our study differ greatly from that reported in previous studies (30.0% and 25.2%, respectively) (Cheng et al., 2020). However, the erythromycin resistance rate of *C. jejuni* was lower than in previous studies (15.4% and 30.1%, respectively), whereas the rate in *C. coli* was higher (41.2% and 18.3%, respectively) (Cheng et al., 2020). We detected high rates of resistance to lincomycin in both *C. jejuni* and *C. coli*, which may be related to the antibiotics commonly used in the areas from which the isolates were collected. The resistance of *C. coli* to antibiotics other than tetracycline and ciprofloxacin was greater than that of *C. jejuni*. These findings are consistent with the results of Tang et al. (Tang et al., 2020a), who reported that the prevalence of antibiotic resistance in chicken-derived *C. coli* was higher than in chicken-derived *C. jejuni*. In general, there is a worrying trend that, although the addition of antibiotics to feed supplements was banned in China in 2020, it has not reduced antibiotic resistance. On the contrary, some antibiotic resistance rates are still rising in some regions (Cheng et al., 2020).

Previous studies have shown that there is a strong correlation between the presence of AMR determinants detected with WGS and phenotypic antibiotic resistance (Rokney et al., 2020; Habib et al., 2023). However, it is known that *Campylobacter* also has many resistance mechanisms other than resistance gene-mediated, such as changes in membrane permeability, modification of the antibiotic efflux pumps, etc. (Iovine, 2013). The determinants of drug resistance do not always confer resistance phenotypes, and single resistance determinant may correlate weakly with certain antibiotics (Šoprek et al., 2022). In this study, we found that the overall correlation between the 16 antibiotic resistance determinants detected with ResFinder v.4.1 and phenotypic resistance was not strong, and that there were huge differences between the different antibiotics. This suggests that current research into the resistance mechanisms of *Campylobacter* remains to be improved, and that simply analyzing bacterial resistance in terms of the antibiotic resistance determinants predicted with WGS does not provide an accurate assessment.

In the present study, phenotypic resistance to ciprofloxacin and tetracycline correlated well with the presence of the *gyrA* gene point mutation (C257T) and the *tet(O)* or *tet(L)* gene, respectively, confirming that they are the main factors conferring resistance to the corresponding antimicrobial agents. CTX-M type β-lactamases usually are the cause of drug resistance of Gram-negative bacteria to cephalosporin such as cefotaxime, but CTX-M was not found in resistant strains in this study. In our isolates, 90% isolates of our study contained *blaOXA*-type β-lactamase-encoding gene. Indeed, most *Campylobacter* strains contain the *bla-OXA* gene encoding β-lactamase that confers resistance to carbapenems, but not to cephalosporin (Hadiyan et al., 2022). Research has already shown that different β-lactamases have different hydrolysis profiles (Poirel et al., 2011) and that the expression of β-lactamase directly affects the resistance of strains to β-lactam antibiotics (Casagrande Proietti et al., 2020). This may also explain why strains containing the *bla-OXA* gene but with a β-lactam-sensitive phenotype have been found in several other studies (Griggs et al., 2009; Zeng et al., 2014; Hadiyan et al., 2022). Our study further confirmed that the presence of *bla-OXA* gene is not related to the resistance of cephalosporin drugs.

The prevalence of aminoglycoside-resistance-related determinants was low in the isolates tested, but these determinants showed relatively high diversity. A previous study demonstrated that the combined action of the *aph(3′)-III*, *aac(6′)-aph(2′′)*, and *ant(6)-Ia* genes conferred resistance to aminoglycoside antibiotics on *Campylobacter* (Zhang et al., 2022), which was confirmed in our study. However, even with the synergistic effect of *ant(6)-Ia* and *aac(6’)-aph(3’’)*, the correlation between each gene and amikacin resistance was still low. Moreover, a *C. coli* isolate containing *aadE-Cc* showed a sensitive phenotype. This finding is consistent with a report by Painset et al. (Painset et al., 2020), who also observed *Campylobacter* strains carrying the *aadE-Cc* gene that were not resistant to some aminoglycoside antibiotics. There may be some unknown mechanism that inactivate these genes in *Campylobacter*.

Erythromycin and lincomycin have similar resistance mechanisms (Zhao et al., 2016; Wang et al., 2022). In the present study, the correlation between *erm*(B) and resistance to those two antibiotics was not strong. Therefore, we analyzed the sequence of 23S rRNA, and found no A2075G mutation.

Since most of the strains in this study have multi-drug resistance, we analyzed the genetic environment of the resistance gene of JZ02 (resistant to six antibiotics), and try to know how this strain obtained the antibiotic resistance gene. As is known that *Campylobacter* can acquire exogenous DNA through natural transformation (Wang and Taylor, 1990). The spread of antibiotic resistance genes in *Campylobacter* isolates from humans, animals, and the environment has previously been reported (Asuming-Bediako et al., 2019). The tetracycline resistance gene *tet(O)* is believed to have originated in Gram-positive cocci (Zilhao et al., 1988), and the tetracycline resistance mediated by this gene is mainly spread via the horizontal transfer of resistance genes on conjugated plasmids (Wardak et al., 2007). Although we found that resistance gene *tet(O)* was located on chromosome of isolate JZ02, some tranposase-encoding sequences were detected near *tet(O)*. The presence of these transposases implies that the antibiotic-resistance genes were co-transferred with some mobile genetic elements into the genomes of related strains. Although no relevant mobile elements were found in cluster 2, several genes, such as *ant(9)* and *aph(2’’)-If*, which encode aminoglycoside-modifying enzymes, are similar to those of some Gram-positive bacteria, indicating that they may have derived from Gram-positive bacteria in the environment or animal intestines (Fabre et al., 2018).

The ability of *Campylobacter* to cause human diseases is considered multifactorial, and several genes are closely related to its virulence, including *ciaB* and *cdtABC* (Lopes et al., 2021). An analysis of the virulence-related genes of our isolates showed that *C. jejuni* carried more virulence-related genes than *C. coli*, which is consistent with the study of Lapierre et al. (Lapierre et al., 2016), and most of these genes were involved in motility (*flaA*), adhesion (*cadF*, *cheA*, *jlpA* et al), and invasion (*ciaB*). It is noteworthy that in this study, CC-21 and CC-464 had the most virulence-related genes and these two clonal complexes are also common among the clinical isolates of *Campylobacter* (Zhang et al., 2020a; Zhang et al., 2020b; Zang et al., 2021). Then we found that the additional genes they carried were mainly involved in immune modulation like bacterial capsule biosynthesis, especially by sugar and aminotransferase enzymes (kfiD, glf, Cj1426c, Cj1432c, Cj1434c, Cj1435c, Cj1436c, Cj1437c) while these genes do not be harbored in other complexes(**Supplementary Table 5**; **Supplementary Figure 2**). Although a high prevalence of virulence-associated genes (*ciaB* and *flaA*) has been already reported in *Campylobacter* strains infecting children with moderate to severe diarrhea (Quetz et al., 2012), these genes were only detected in *C. jejuni* in the present study. This may explain why *C. jejuni* colonizes its host more readily than *C. coli* and is responsible for more food-borne bacterial infection events (Moffatt et al., 2019; Callahan Sean et al., 2021; Schirone and Visciano, 2021). Virulence genes related to the type IV secretion system were only found in one strain of *C. jejuni*, and these genes are less prevalent in Asia and Europe (Panzenhagen et al., 2021). We also detected the *wlaN* gene, which is involved in Guillain–Barre syndrome in two *C. jejuni* isolates(Guirado et al., 2020).

In conclusion, in this study, we have demonstrated the genetic diversity and antimicrobial susceptibility of *Campylobacter* isolated from local dual-purpose chickens in China, and analyzed their resistance- and virulence-related genes. It thus provides important data on the epidemiological characteristics of *Campylobacter* in this food source.

## Data availability statement

The datasets presented in this study can be found in online repositories. The names of the repository/repositories and accession number(s) can be found in the article/**Supplementary Material**.

## Ethics statement

The studies involving animals were reviewed and approved by the Ethics Committee of Institute of Animal Husbandry and Veterinary, Hubei Academy of Agricultural Sciences (Wuhan, China).

## Author contributions

JX, YC, QingL, and TZ conceived and designed the experiments. JX, YC, QinL, and YG performed the experiments. GW, WZ, and QH analyzed the data. JX, HS, and TZ wrote the manuscript. All authors contributed to the article and approved the submitted version.

## Supplementary material

Supplementary Figure S1 Matrix of SNP pair counts among 30 isolates. Number of SNP was calculated by comparing the genome sequences to the reference (RM1221) genome. Background colors represent different number of pairwise SNPs.

Supplementary Figure S2 Distribution of virulence-related genes of 30 isolates.
