# Supplementary figures and images for: Genetic characteristics, antimicrobial susceptibility, and virulence genes distribution of Campylobacter isolated from local dual-purpose chickens in central China
Source: Front Cell Infect Microbiol. 2023 Sep 7;13:1236777. doi: 10.3389/fcimb.2023.1236777 (PMC10517862; doi:10.3389/fcimb.2023.1236777)

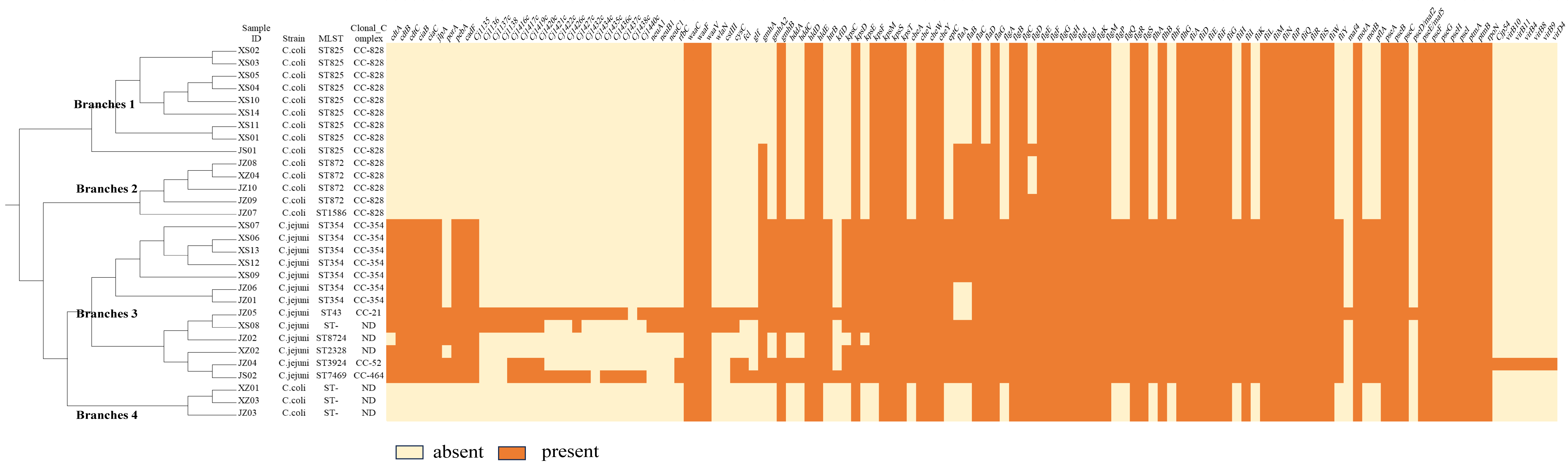

Supplement: Supplementary Figure S1 — Matrix of SNP pair counts among 30 isolates. Number of SNP was calculated by comparing the genome sequences to the reference (RM1221) genome. Background colors represent different number of pairwise SNPs. [file Image_1.tif]

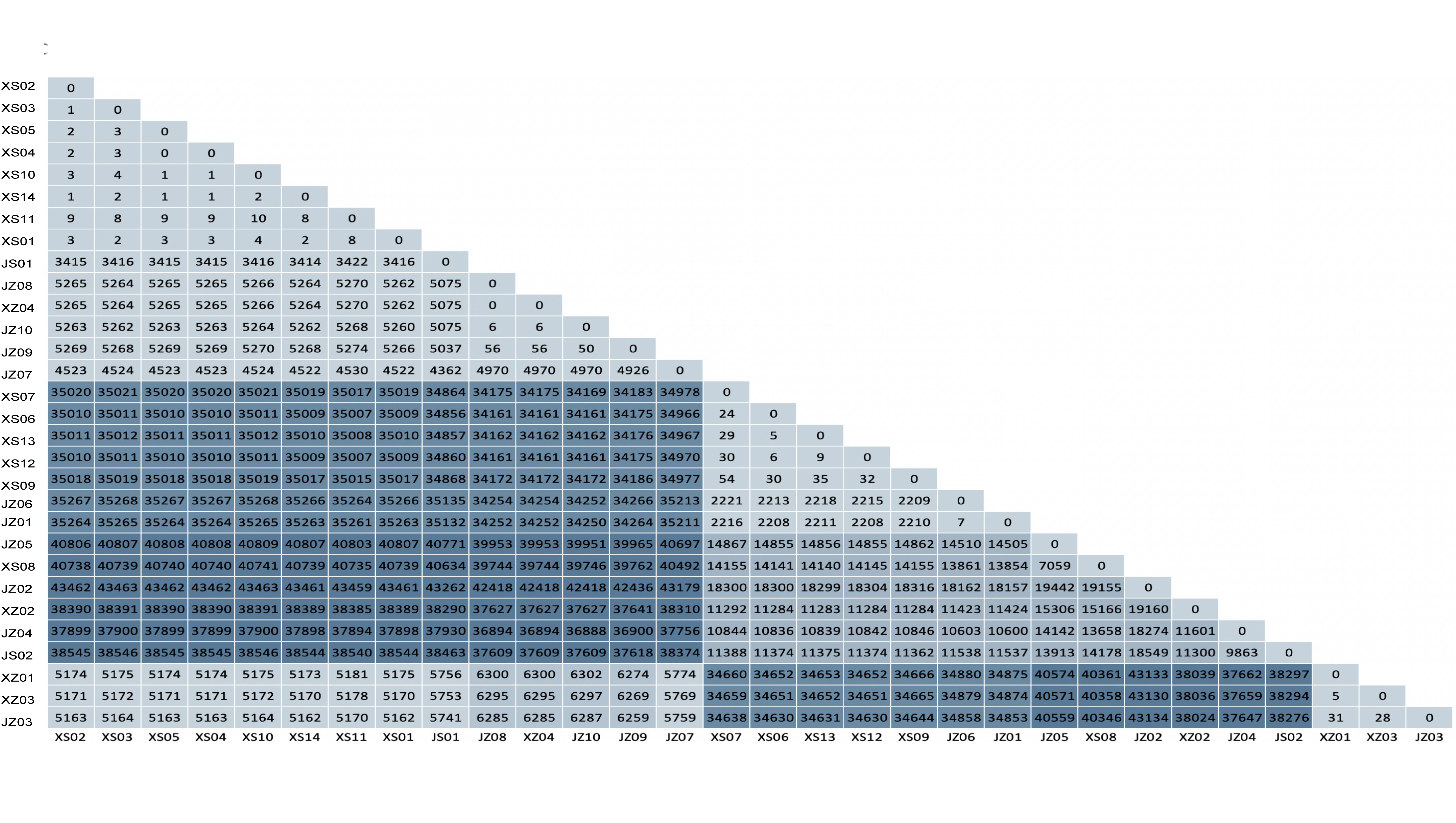

Supplement: Supplementary Figure S2 — Distribution of virulence-related genes of 30 isolates. [file Image_2.tif]
